# Novel screening assay for *in vivo* selection of *Klebsiella pneumoniae* genes promoting gastrointestinal colonisation

**DOI:** 10.1186/1471-2180-12-201

**Published:** 2012-09-11

**Authors:** Erik J Boll, Lene N Nielsen, Karen A Krogfelt, Carsten Struve

**Affiliations:** 1 Department of Microbiology and Infection Control, Statens Serum Institut, DK-2300, Copenhagen, Denmark; 2 WHO Collaborating Centre for Reference and Research on Escherichia and Klebsiella, Statens Serum Institut, DK-2300, Copenhagen, Denmark; 3 Present address: Department of Veterinary Pathobiology, Faculty of Life Sciences, University of Copenhagen, Frederiksberg, Denmark

**Keywords:** *Klebsiella pneumoniae*, Genomic library, Mouse model of gastrointestinal colonisation

## Abstract

**Background:**

*Klebsiella pneumoniae* is an important opportunistic pathogen causing pneumonia, sepsis and urinary tract infections. Colonisation of the gastrointestinal (GI) tract is a key step in the development of infections; yet the specific factors important for *K. pneumoniae* to colonize and reside in the GI tract of the host are largely unknown. To identify *K. pneumoniae* genes promoting GI colonisation, a novel genomic-library-based approach was employed.

**Results:**

Screening of a *K. pneumoniae* C3091 genomic library, expressed in *E. coli* strain EPI100, in a mouse model of GI colonisation led to the positive selection of five clones containing genes promoting persistent colonisation of the mouse GI tract. These included genes encoding the global response regulator ArcA; GalET of the galactose operon; and a cluster of two putative membrane-associated proteins of unknown function. Both ArcA and GalET are known to be involved in metabolic pathways in *Klebsiella* but may have additional biological actions beneficial to the pathogen. In support of this, GalET was found to confer decreased bile salt sensitivity to EPI100.

**Conclusions:**

The present work establishes the use of genomic-library-based *in vivo* screening assays as a valuable tool for identification and characterization of virulence factors in *K. pneumoniae* and other bacterial pathogens.

## Background

*Klebsiella pneumoniae* is an important cause of opportunistic infections, such as pneumonia, sepsis and urinary tract infections [1]. Studies also link *K. pneumoniae* infections to inflammatory bowel diseases as well as liver abscesses [2-5]. Moreover, multiresistant strains are frequently observed, stressing the need to find new ways to prevent and treat *K. pneumoniae* infections [6-8].

Characteristically, most *K. pneumoniae* infections are preceded by colonisation of the patients gastrointestinal (GI) tract which is also considered the main reservoir for transmission of the pathogen [9,10]. In order to persist in this extremely competitive environment, any invading pathogen must be able to compete with the indigenous microbiota for nutrients, grow at a rate sufficient to avoid washout, or, alternatively, adhere to the mucosal surface [11]. The specific factors important for the ability of *K. pneumoniae* to colonize and reside in the GI tract of the host are largely unknown. The presence of an urease has been shown to be important for GI colonisation by the pathogen [12], as well as lipopolysaccharide (LPS) [13], whereas studies on the role of capsule are contradictory [14,15]. It is to be expected that other still unknown factors are required for *K. pneumoniae* to colonize and reside in the GI tract. An increased knowledge of such factors is an important step in the search for new strategies to prevent colonisation and subsequent infection of susceptible patients with *K. pneumoniae*.

One approach to identify novel pathogenic virulence mechanisms is to employ screening of genomic libraries. Such libraries are constructed by digesting genomic DNA, cloning it into vectors and transforming them into cells that can be screened for a desired phenotype [16-19]. In a previous study, we constructed a library of *K. pneumoniae* DNA expressed in *Escherichia coli* and successfully used it to screen for *K. pneumoniae* genes involved in biofilm formation *in vitro*[18].

The objective of this study was to identify genes involved in *K. pneumoniae* intestinal colonisation by screening of the *K. pneumoniae* genomic library in a well-established mouse model of GI colonisation. To our knowledge, this is the first use of a genomic library as a positive-selection-based *in vivo* screening model. We demonstrate successful *in vivo* selection of clones containing GI colonisation promoting *K. pneumoniae* genes, thus validating this novel screening approach.

## Results

### Clones containing colonisation promoting genes are selected in the mouse GI colonisation model

**Figure 1:**
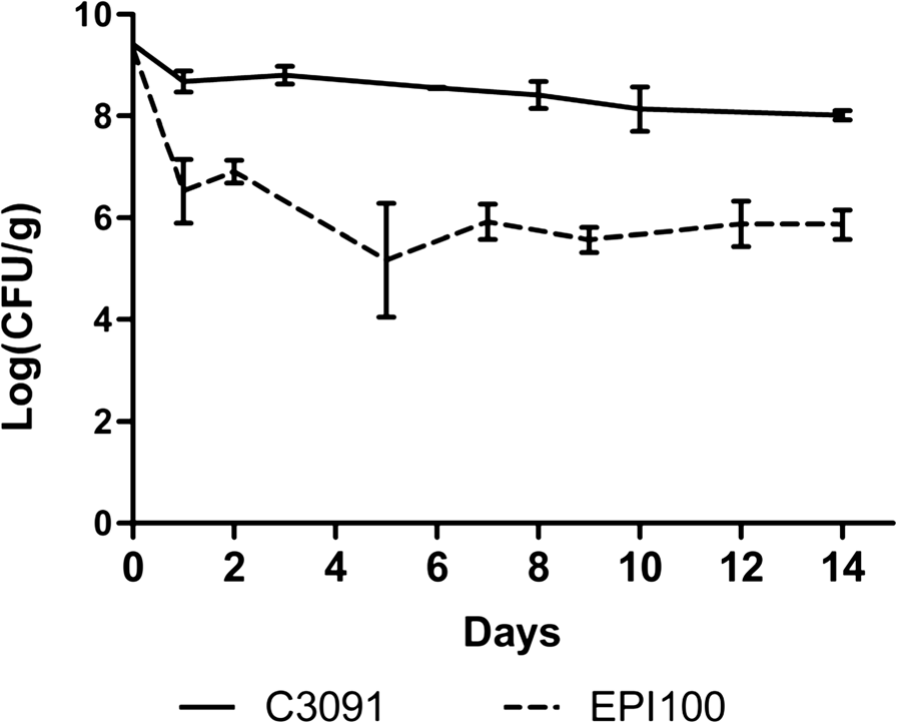
**Colonisation of the intestine by*K. pneumoniae*C3091 and*E. coli*EPI100. The two strains were fed individually to sets of three mice.** Colonisation was quantified from plating of faeces on selective media. Symbols for day 0 represent the size of inoculum. The results are presented as the mean log (CFU/g faeces) ± sum of means (SEM).

We initially assessed the colonisation abilities of *K. pneumoniae* clinical isolate C3091 and *E. coli* laboratory strain EPI100 in the mouse model of GI colonisation. We found that while both strains persistently colonized the intestines of the infected mice, the bacterial counts in faeces were more than 100-fold higher for C3091 than for EPI100 (Figure 1). Thus *K. pneumoniae* C3091 is a superior coloniser of the intestinal tract likely via possession of genes not present in the *E. coli* strain and which promote enhanced colonisation ability.

**Figure 2:**
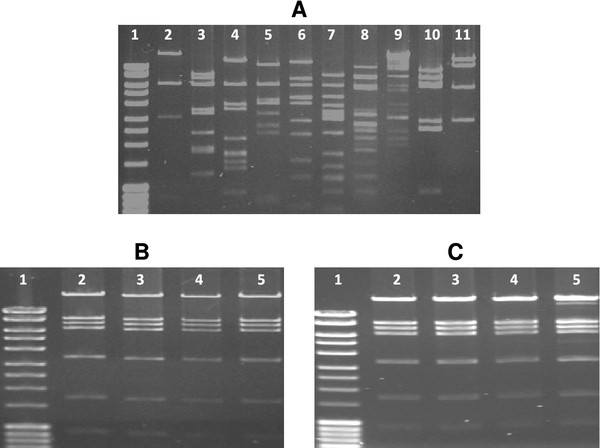
**Specific fosmid clones are selected during intestinal colonisation.** Restriction enzyme analysis of fosmid pools before and after inoculation into mice. 10 colonies were randomly picked from plating of the inoculum fed to two mice on day 0 (**A**, lanes 2–11). On day 17 postfeeding, 4 colonies were picked from plating of faeces from each of the two mice (**B** and **C**, lanes 2–5). Fosmids were isolated and cut with restriction enzyme *Sal*I. The presented data (shown here for fosmid pool 1) are representative for all 12 fosmid pools.

To identify GI colonisation promoting genes, a library consisting of 1,152 fosmids, each containing approximately 40 kb random *K. pneumoniae* C3091 DNA, expressed in *E. coli* EPI100 was screened in the mouse GI colonisation model. The library was arrayed in 12 pools each containing 96 fosmid clones. The 12 pools were fed individually to a set of two mice, and following 17 days of colonisation, fosmids were purified from colonies picked from platings of faecal samples and characterised. The 17-day colonisation period was chosen to ensure enough time for detectable selection of clones containing colonisation promoting genes. Indeed, restriction enzyme analysis revealed that from each pool only a single fosmid clone was present in high numbers in the faeces of mice originally inoculated with 96 individual clones (Figure 2). Thus a striking selection had occurred in the mouse intestine, indicating that the selected clones contain *K. pneumoniae* genes promoting GI colonisation.

Restriction enzyme analysis and partial sequencing of the *in vivo* selected clones revealed that some of the clones contained overlapping inserts of C3091 DNA. As the GI colonisation promoting genes among these clones were expected to be identical, one clone from each group of clones with overlapping inserts was selected. Thus a total of five clones were further characterised (hereon referred to as clones 1–5).

**Figure 3:**
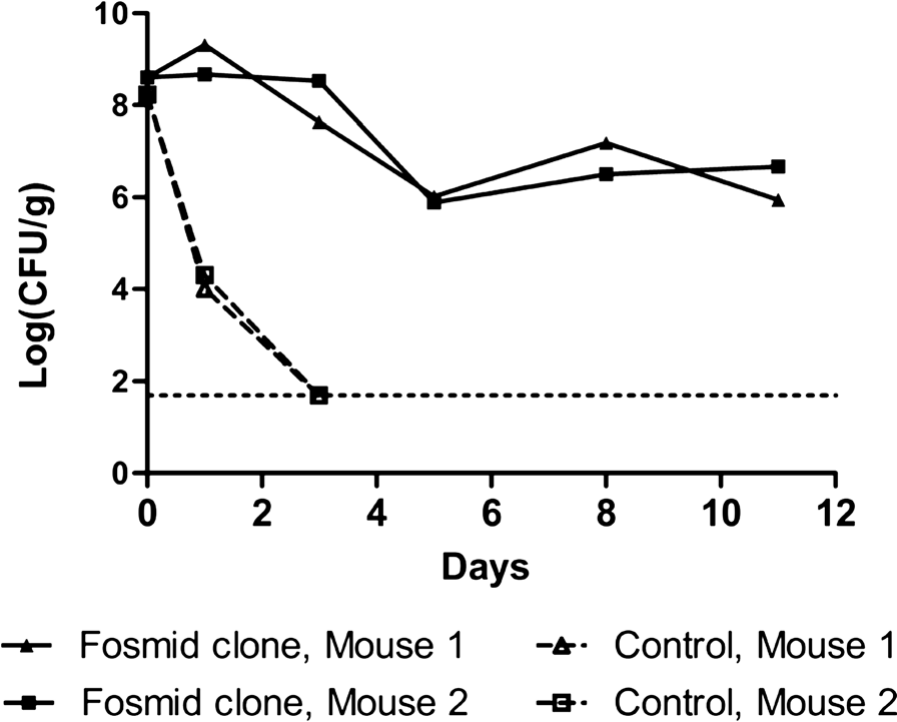
**The selected*K. pneumoniae*C3091-derived fosmids confer enhanced GI colonisation to EPI100.** The ability of each EPI100 fosmid clone (filled symbols) to outcompete EPI100 carrying the empty pEpiFOS vector (open symbols) was tested by feeding sets of two mice with equal amounts of the control strain and one of the fosmid clones. The presented data is for fosmid clone 2. Three days post-feeding, the bacterial counts of the control strain were below the detection limit of 50 CFU/g faeces (dashed horizontal line). Similar results were obtained for all fosmid clones.

We then sought to confirm the presence and expression of *K. pneumoniae* C3091 genes promoting GI colonisation in the five selected clones. In separate experiments, each clone was fed to two mice simultaneously with EPI100 carrying the empty fosmid vector. All five clones displayed markedly increased colonisation ability and rapidly outcompeted the EPI100 vector control strain, thereby verifying the acquisition of colonisation promoting *K. pneumoniae* genes (Figure 3).

It could be speculated that the enhanced GI colonisation abilities of the selected clones was due to a generally enhanced growth rate. To test this, each of the five clones were evaluated for their ability to outgrow EPI100 carrying the empty fosmid vector when grown competitively in LB broth. Four of the clones grew to the same level as the control strain. However, the bacterial counts for the fifth clone were a 100-fold higher than the control strain at the end of the *in vitro* growth experiment, indicating that the *K. pneumoniae* genes present in this particular clone have a general growth promoting effect.

### Identification of the *K. pneumoniae* genes promoting enhanced GI colonisation

Each fosmid contains approximately 40 kb of *K. pneumoniae* DNA. Thus, to identify the specific GI colonisation promoting genes, a library of 96 subclones, containing 4–12 kb C3091 DNA fragments inserted into cloning vector pACYC184, were constructed from each of the five fosmid clones. The subclones within each library were then pooled and fed to a set of three mice in separate experiments. Following 5–7 days of infection, plasmids from stool samples were isolated and submitted to *Sal*I digestion profiling. While we were unable to obtain clonal selection from the subclone library derived from fosmid clone 5, we successfully observed selection of a single clone in each of the four other experiments (data not shown). The colonisation promoting abilities of the C3091 DNA fragments in these four subclones were verified in the mouse model in pair-wise growth-competition experiments against EPI100 carrying the empty pACYC184 vector. Each of the four selected subclones retained the GI colonisation advantage of the respective fosmid clones from which they were derived (data not shown), thus once again confirming the acquisition of GI colonisation promoting genes.

**Figure 4:**
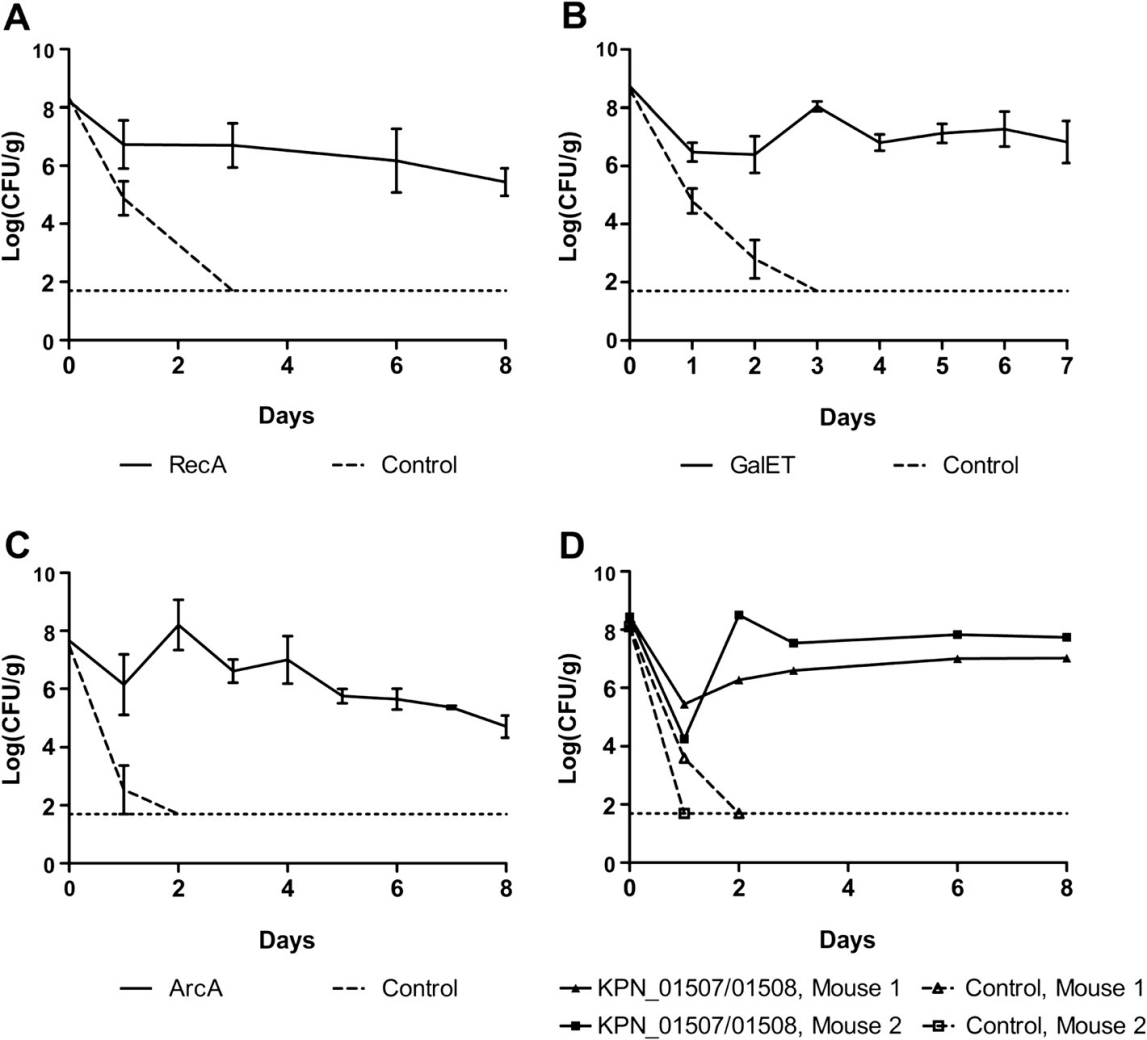
***K. pneumoniae*C3091-derived RecA, GalET, ArcA and putative proteins KPN_01507/01508 confer enhanced GI colonisation to EPI100.** Sets of mice were fed with equal amounts of EPI100 carrying the empty pACYC184 vector and EPI100 carrying pACYC184-*recA*, -*galET*, -*arcA*, or –*kpn_01507/01508*, respectively. In all four experiments, the bacterial counts of the control strain were below the detection limit of 50 CFU/g faeces (dashed horizontal lines) one-to-three days post-feeding. The data in Figure 4**A**-**C** are expressed as the mean ± SEM for three infected mice.

We next sequenced the C3091 DNA fragments of the four selected subclones. Based on these sequences, clones containing only a single C3091 gene or gene cluster were constructed by PCR amplification using specific primers and insertion into pACYC184. These well-defined clones were tested in the mouse model in competition experiments against EPI100 carrying the empty PACYC184 vector (Figure 4). This successfully led to identification of the genes from each of the fosmid clones encoding colonisation promoting *Klebsiella* proteins. These were: the RecA recombinase; UDP-galactose-4-epimerase (GalE) and galactose-1-phosphate uridylyltransferase (GalT) of the galactose operon; the ArcA response regulator; and a cluster of two hypothetical proteins homologous to KPN_01507 and KPN_01508 in the sequenced genome of *K. pneumoniae* strain MGH78578 and encoding proteins of unknown function. Sequence analysis showed that all six proteins share 99-100% identity with their corresponding homologues in MGH78578. EPI100 carrying pACYC184 with either of these genes or gene clusters outcompeted the corresponding vector control strain within 3 days and persisted in the mouse intestines throughout the experiments (Figure 4).

### GalET confer decreased sensitivity to bile salts

**Figure 5:**
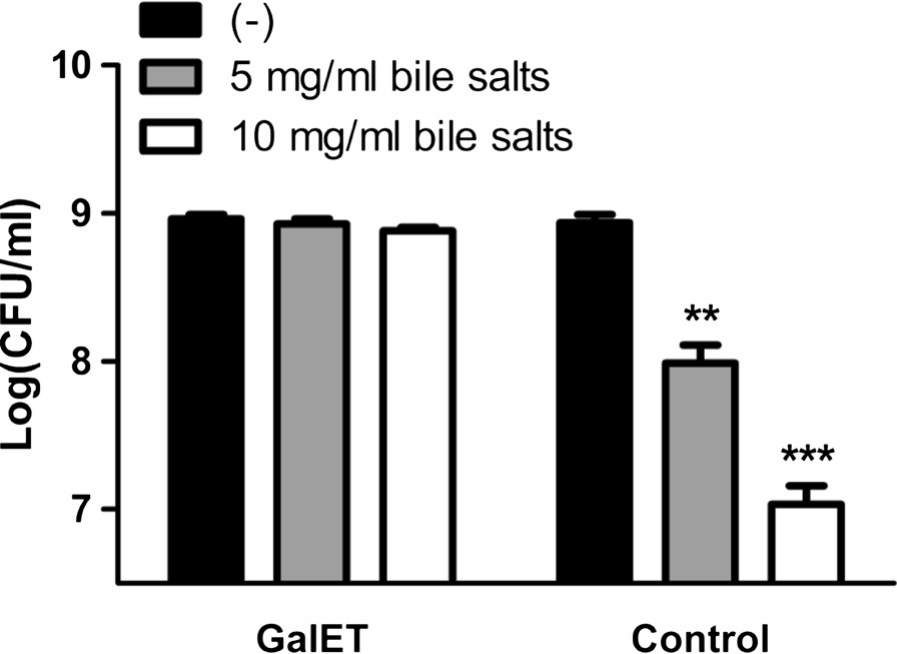
***K. pneumoniae*C3091-derived GalET confer decreased sensitivity to bile salts to*E. coli*EPI100.** EPI100 carrying either pACYC184-*galET* or the pACYC184 vector control were grown for 18 hrs in LB broth in the presence and absence of increasing concentrations of bile salts after which colonisation was quantified from plating. The data are expressed as the mean ± SEM for triplicate samples. ***, *p* < 0.001; **, *p* < 0.01, as compared to untreated EPI100 vector control.

We thought to characterise directly the growth enhancing properties of the C3091-derived *galET* genes. We found that EPI100 carrying pACYC184-*galET* failed to ferment galactose *in vitro* (data not shown), suggesting that the colonisation enhancing effect is not attributable to galactose fermentation. However, the GalETKM operon also plays a key role in modifying galactose for assembly into LPS [20], and mutations in LPS synthesis genes have been shown to attenuate the survival of *E. coli* strain MG1655 in the mouse intestine, partly due to enhanced susceptibility to bile salts [21]. Intriguingly, EPI100 carrying pACYC184-*galET* demonstrated clearly decreased sensitivity to bile salts *in vitro* compared to the EPI100 vector control strain (Figure 5). These findings suggest that the C3091-derived *galET* genes confer enhanced colonisation abilities to EPI100 in the mouse model by decreasing the sensitivity of the strain to bile salts.

## Discussion

Colonisation of the GI tract plays a key role in the ability of *K. pneumoniae* to cause disease, stressing the need for an increased understanding of the mechanisms underlying this important feature. In this study, we employed a genomic-library-based approach to identify *K. pneumoniae* genes promoting GI colonisation. We demonstrated that screening of a *K. pneumoniae* C3091-based fosmid library, expressed in *E. coli* strain EPI100, in a mouse model led to the positive selection of clones containing genes which promote GI colonisation. Thus, oral ingestion of pooled library fosmid clones led to a successful selection of single clones capable of persistent colonisation of the mouse GI tract. This is a testament to the remarkably competitive environment of the GI tract where only clones having obtained a colonisation advantage will be able to colonise and persist in high numbers due to the presence of the endogenous microflora. When tested individually in growth competition experiments against EPI100 carrying the empty fosmid vector, each of the selected fosmid clones rapidly outcompeted the control strain. Based on these clones, we were able to identify C3091 genes and gene clusters conferring enhanced GI colonisation, including *recA*, *galET* and *arcA*.

Notably, EPI100 harbours deletions in *recA*, suggesting that the selection of *K. pneumoniae* C3091-derived *recA* reflects complementation of this missing *E. coli* gene. RecA plays an essential role in chromosomal recombination and repair, and *E. coli* RecA mutant strains have been shown to exhibit attenuated growth rates *in vitro*[22,23]. Indeed, EPI100 carrying pACYC184-*recA* also showed a clear growth advantage compared to the vector control when grown in LB broth. This finding verifies that RecA plays a significant role in bacterial growth in general and thus the GI colonisation promoting effect of *recA* is most likely due to a generally enhanced growth rate of the *recA* containing clone. Nevertheless, while the selection of RecA in the mouse model is not a surprising finding it serves as a proof of principle, regarding the validity of the screening approach.

The fact that pACYC184-*galET* was unable to ferment galactose *in vitro* was to be expected since EPI100 harbours deletions in galactokinase (GalK) and UTP-glucose-1-phosphate uridylyltransferase (GalU), both of which are necessary for growth on galactose [24-26]. Instead, we observed an intriguing decreased sensitivity to bile salts *in vitro* conferred by C3091-derived GalET*.* Further studies are needed to characterise the mechanism underlying this phenotype and its physiological implications. However, we speculate that incorporation of C3091 GalET-mediated sugar-residues into the bacterial membrane, i.e. as a part of LPS as previously described [20], may have an enhancing effect on the membrane stability, thus promoting decreased sensitivity to bile salts and possibly other compounds such as antimicrobial peptides present in the mouse GI tract. In support of this, enterohaemorrhagic *E. coli gal* mutant strains have been shown to be 500-fold less able to colonise the GI tract of rabbits and 100-fold more susceptible to antimicrobial peptides than the parent strain [26].

Together with the sensor transmitter protein ArcB, ArcA constitutes a two-component ArcAB system which functions as a global regulator of genes involved in metabolism in response to oxygen availability, primarily favouring anaerobic growth [27]. ArcA homologues have, moreover, been implicated in regulating the expression of virulence factors and proteins involved in serum resistance [28,29]. To our knowledge, the EPI100 strain does not harbour mutations in ArcAB, thus indicating a cumulative effect of native and *K. pneumoniae*-derived ArcA activity promoting enhanced colonisation. To assess whether this effect was due to enhanced adaption to anaerobic growth in general, we tested EPI100 carrying pACYC184-*arcA* for its potential enhanced ability to grow under anaerobic conditions in LB broth in competition with the EPI100 vector control. We did not observe any significant differences in the growth rate between the two strains. Thus, although a growth promoting effect of ArcA in the intestinal environment cannot be excluded from these *in vitro* assays, the effect of ArcA on GI colonisation may instead be via the regulation of colonisation factors not related specifically to anaerobic growth. Notably, during screening of a *K. pneumoniae* C3091 mutant library we previously found an *arcB* transposon mutant to be markedly attenuated in GI colonisation [13]. The identification of the *arcAB* regulon by two fundamentally different screening approaches emphasizes the key role of ArcAB in GI colonisation and furthermore underscores the validity of the screening approaches.

Our screening assay also identified a *Klebsiella* two-gene cluster of unknown function, here designated *kpn_01507* and *kpn_01508*, which conferred enhanced GI colonisation ability to EPI100. KPN_01507 is a putative membrane protein, whereas the use of SignalP 4.0 predicted the presence of a secretory signal peptide in KPN_01508, a signal targeting its passenger domain for translocation across the bacterial cytoplasmic membrane [30]. These findings, therefore, suggest that KPN_01508 may be translocated and/or secreted from the cell. Interestingly, homologues of both genes are found among several sequenced strains of *K. pneumoniae* but do not appear to be present in *E. coli*. Future studies may reveal the function of these genes in GI colonisation.

The fact that genes associated with metabolism were selected in the *in vivo* screening assay is not surprising since the ability to obtain nutrients for growth is essential for any GI colonizing organism. However, many highly conserved proteins involved in metabolism are increasingly recognized as having additional roles, some of which are related to bacterial virulence [31]. The GalET cluster may be viewed as an example of such so-called moon-lighting proteins as the colonisation enhancing effect was not associated with galactose fermentation per se but was due to increased resistance against bile salt possibly mediated by the modification of LPS core synthesis.

A key limitation of the library-based technique is its inability to identify interactions among distant genetic loci. This limitation could be circumvented by using co-expressed plasmid- and fosmid-based genomic libraries as recently described [16]. Thus, future studies combining the C3091 fosmid library with a co-expressed plasmid-based C3091 library may lead to the selection of more GI-enhancing genes than those obtained in this study.

The fact that our screening method is based on a laboratory *E. coli* strain, as opposed to a commensal *E. coli* isolate, raises another important point. Genes mutated in the laboratory strain, e.g. *recA*, would most likely not have been selected if the screening had been carried out using a commensal strain. However, since commensal *E. coli* are already excellent GI colonisers, it is possible that genes which are important for *K. pneumoniae* GI colonisation but also present in *E. coli* commensal strains will not be selected in the screening. However, if the objective is to specifically identify *K. pneumoniae* virulence genes, using a commensal *E. coli* strain as a host in the screening will be a favourable approach.

Using *E. coli* as a host has several advantages when it comes to construction, cloning, and expression of the fosmid library. However, a shortcoming is that although the assay identifies genes promoting colonisation in *E. coli*, additional studies involving the construction of specific mutants are warranted to verify the role of these genes in *K. pneumoniae*. Although future studies are needed to characterise the role of *galET* and *kpn*_*01507/01508* in *K. pneumoniae* colonisation, as discussed above, both *recA* and *arcA* are expected to play a significant role in *K. pneumoniae* colonisation.

## Conclusions

A novel screening approach to identify genes involved in GI colonisation was successfully applied. Thus, by screening a clone library of a *K. pneumoniae* genome for enhanced GI colonisation abilities in a mouse model, a selection of single clones containing GI colonisation promoting genes was obtained. The methodology was validated as *K. pneumoniae* genes complementing deleted genes in the *E. coli* EPI100 background and genes previously identified to promote GI colonisation were selected in the assay. Furthermore, previously unrecognized genes involved in GI colonisation were identified. Moreover, our findings demonstrate the usefulness of this screening approach for the identification of genes involved in metabolic pathways and that these genes may have additional biological actions beneficial to the pathogen.

The methodology can easily be adapted to other bacterial pathogens and infection models. Thus *in vivo* screening of genomic libraries may be a valuable tool for future studies to identify and characterise virulence factors in bacterial pathogens.

## Methods

### Bacterial strains and growth conditions

The following two streptomycin-resistant strains were used: C3091, a clinical *K. pneumoniae* isolate from a patient with urinary tract infection [32]; and EPI100 [F– *mcr*A Δ(*mrr-hsd*RMS-*mcr*BC) ϕ80d*lac*ZΔM15 Δ*lac*X74 *rec*A1 *end*A1 *ara*D139 Δ(*ara, leu*)7697 *gal*U *gal*K λ– *rps*L *nup*G *ton*A], a laboratory *E. coli* strain (Epicentre).

The genomic library consists of 1,152 *E. coli* EPI100 clones, each carrying a fosmid containing approximately 40 kb of *K. pneumoniae* C3091 DNA as previously described [18].

Bacteria were routinely cultured in Luria-Bertani (LB) broth or on LB or MacConkey agar plates containing the following antibiotics where appropriate: 30 μg/ml chloramphenicol, 25 μg/ml kanamycin, and 100 μg/ml streptomycin.

### Mouse model of GI colonisation

Six- to eight-week old female outbred CFW1 (Harlan) mice were used for GI colonisation experiments as previously described [15,33]. Briefly, mice were caged in groups of two or three and given sterile water containing 5 g/L streptomycin sulphate throughout the experiment. Streptomycin treatment selectively removes facultative anaerobes while leaving the anaerobic microbiota essentially intact [34]. After 24 h, 100 μl bacterial suspensions of approximately 10^9^ colony forming units (CFU) were administered orally. Faeces were collected every second or third day, homogenised in 0.9% NaCl, and serial dilutions were plated on selective media. None of the inoculated mice developed any symptoms during the colonisation period. All animal experiments were conducted under the auspices of the Danish Animal Experiments Inspectorate, the Danish Ministry of Justice.

### Construction of subclone libraries

Purified fosmids were submitted to partial digestion with *Bfu*CI, after which ~4-12 kb DNA fragments were excised and purified from low-melting point agarose gels, and then ligated into the *Bam*HI site of pACYC184 and transferred to *E. coli* EPI100. EPI100 subclones were selected by growth on LB plates containing 30 μg/ml chloramphenicol.

### Cloning of fosmid-derived colonisation promoting *K. pneumoniae* C3091 genes

*G*enes or gene clusters were PCR amplified from the *K. pneumoniae* C3091 gene fragments of the respective selected fosmid-derived subclones. All primers used, and the restriction sites introduced at their 5’ ends, are listed in Table 1. The PCR products were digested with the respective restriction enzymes and ligated into the corresponding sites of pACYC184.

**Table 1:** **Primers used in this study for construction of plasmids encoding colonisation promoting*K. pneumoniae*C3091 genes**

**Primer**	**Sequence (5’ → 3’)**^**a**^
*recA*-*Bsp*HI	GCGCGCTCATGACGGAGCGGCGTGATGAAGG
*recA*-*Hind*III	GCGCGCAAGCTTAAATATTAACGGCGAAGCGAACAC
*arcA*-*Bsp*HI	GCGCGCTCATGATCGCGTGGACTGGTATGC
*arcA*-*Hind*III	GCGCGCAAGCTTTGAGCCGGGTAAAGATTGTGACTA
*kpn_01507*-*Bsp*HI	GCGCGCTCATGAGCAATGACCGCCGGGACAGGAG
*kpn_01508*-*Hind*III	GCGCGCAAGCTTTCTAGGATCGCCGGCACAATAATG

^a^ Restriction sites highlighted by underscoring.

### Bile salt sensitivity assay

Overnight cultures were diluted 1:1000 in LB broth in the absence and presence of various concentrations of Bile Salts no. 3 (Difco) and incubated ~18 hrs at 37°C with shaking. The cultures were then diluted 1:10 in LB broth after which serial dilutions were plated.

### Statistical analysis

Student’s *t*-test was used for statistical evaluation and *p* values < 0.05 were considered statistically significant.

## Competing interests

The authors declare that they have no competing interests.

## Authors’ contributions

EJB participated in the study design, carried out laboratory work, analysed the data, and drafted the manuscript. LNN participated in the study design, carried out laboratory work, analysed the data, and edited the manuscript. KAK participated in the study design, edited the manuscript, and received the funding needed to complete the research. CS conceived the study, carried out laboratory work, analysed the data, and edited the manuscript. All authors have read and approved the final manuscript.
